# Transgender Identity Is Associated With Bullying Involvement Among Finnish Adolescents

**DOI:** 10.3389/fpsyg.2020.612424

**Published:** 2021-01-08

**Authors:** Elias Heino, Noora Ellonen, Riittakerttu Kaltiala

**Affiliations:** ^1^Faculty of Medicine and Health Technology, Tampere University, Tampere, Finland; ^2^Faculty of Social Sciences, Tampere University, Tampere, Finland; ^3^Department of Adolescent Psychiatry, Tampere University Hospital, Tampere, Finland; ^4^Vanha Vaasa Hospital, Vaasa, Finland

**Keywords:** transgender, minority, non-conforming, victimization, bullying

## Abstract

**Background:**

During adolescence, bullying often has a sexual content. Involvement in bullying as a bully, victim or both has been associated with a range of negative health outcomes. Transgender youth appear to face elevated rates of bullying in comparison to their mainstream peers. However, the involvement of transgender youth as perpetrators of bullying remains unclear in the recent literature.

**Objective:**

The aim of this study was to compare involvement in bullying between transgender and mainstream youth and among middle and late adolescents in a general population sample.

**Methods:**

Our study included 139,829 students in total, divided between a comprehensive school and an upper secondary education sample. Associations between gender identity and involvement in bullying were first studied using cross-tabulations with chi-square statistics. Logistic regression was used to study multivariate associations. Gender identity was used as the independent variable, with cisgender as the reference category. Subjection to and perpetration of bullying were entered each in turn as the dependent variable. Demographic factors, family characteristics, internalizing symptoms, externalizing behaviors, and involvement in bullying in the other role were added as confounding factors. Odds ratios (OR) with 95% confidence intervals (95% CI) are given. The limit for statistical significance was set at *p* < 0.001.

**Results:**

Both experiences of being bullied and perpetrating bullying were more commonly reported by transgender youth than by cisgender youth. Among transgender youth, all involvement in bullying was more commonly reported by non-binary youth than those identifying with the opposite sex. Logistic regression revealed that non-binary identity was most strongly associated with involvement in bullying, followed by opposite sex identity and cisgender identity. Transgender identities were also more strongly associated with perpetration of bullying than subjection to bullying.

**Conclusion:**

Transgender identity, especially non-binary identity, is associated with both being bullied and perpetrating bullying even when a range of variables including internal stress and involvement in bullying in the opposite role are taken into account. This suggests that bullying during adolescence may serve as a mechanism of maintaining heteronormativity.

## Introduction

Gender identity refers to an individual’s innate sense of being male, female or an alternative gender (Bockting, 1999). Distinct from gender identity, gender expression refers to an individual’s various characteristics which, during a given period, are generally viewed as masculine or feminine (Coleman et al., 2012). While various gender identities exist, the vast majority of individuals present with cisgender identity, meaning that their gender identity is aligned with their birth-assigned sex. Gender minorities are individuals whose gender identity differs to various degrees from their birth-assigned sex. We refer to all gender minorities as transgender. This encompasses those who identify with the opposite sex and those whose gender identity aligns with both or neither sex or varies (non-binary or gender non-conforming gender identity).

Bullying is defined as aggressive behavior in which a pupil or group of pupils intentionally harm victims in various ways, usually over a period of time, and is usually characterized by a power imbalance between the victim and the bully or bullies (Olweus, 1993; King et al., 1996). Bullying may assume various forms, such as physical violence, verbal abuse, spreading rumors, exclusion from peer groups or then sexual gestures or remarks (Olweus, 2013). Cyberbullying extends the scope of bullying to various information technologies, such as social media and mobile phones (Lindfors et al., 2012). A considerable share of bullying among adolescents is of a sexual nature (Ashbaughm and Cornell, 2008) and often refers scornfully to homosexuality and gender-non-conforming self-expression (Toomey et al., 2012).

Adolescents’ involvement in bullying is common (Kaltiala-Heino and Fröjd, 2011; Lessne et al., 2016), and whether this is as a victim or as a perpetrator, it has well documented negative associations with health and educational trajectories (Chan and Wong, 2015a). Being bullied has been associated, for example, with depression and suicidal ideation (Liang et al., 2007; Kaltiala-Heino and Fröjd, 2011; Heikkilä et al., 2013) and school truancy and impaired academic performance (Wormington et al., 2016). Being a bully has likewise been associated with depression (Klomek et al., 2008; Kaltiala-Heino et al., 2009; Kaltiala-Heino and Fröjd, 2011) and suicidal ideation (Kaltiala-Heino et al., 1999; Heikkilä et al., 2013), but also with delinquency and substance abuse (Liang et al., 2007).

An abundance of research suggests that sexual minority youth report being bullied 1.5–2 times more commonly than mainstream youth (Friedman et al., 2011; Abreu and Kenny, 2018; Kurki-Kangas et al., 2019; McKay et al., 2019). Recent research has also begun to unveil disparities in bullying involvement between gender minority and cisgender youth, particularly regarding disparities in being bullied. US-based research indicates that transgender youth, in school samples, are bullied more often than their cisgender peers (Day et al., 2018; Eisenberg et al., 2019; Johns et al., 2019; Bishop et al., 2020). Transgender youth have been reported to more commonly experience bullying related to gender or sexual orientation (Day et al., 2018) but also bullying related to weight and size (Bishop et al., 2020). In a clinical UK-based sample, almost 90% of transgender youth reported being bullied (Witcomb et al., 2019).

Both sexual minority and transgender youth may differ from the mainstream by gender expression not conforming to traditional male and female roles (i.e., for males by being feminine or for females by being masculine), which could render them susceptible to being bullied, a behavior commonly directed at peers perceived as different (Jones et al., 2018; Price-Feeney et al., 2018). When comparing birth-assigned males and females in a school sample, Lowry et al. (2020) found that youth who described their appearance as gender non-conforming (i.e., males believing they were perceived as feminine or females believing they were perceived as masculine) were violently victimized more often than those youth who described themselves as gender conforming and that the association was stronger among male students. van Beusekom et al. (2020) likewise found that gender non-conformity was associated with general victimization and homophobic name calling and that the associations were stronger among males. Further, among transgender youth, those who perceived themselves as gender non-conforming were bullied more frequently than those transgender youth who perceived themselves as gender conforming, and also within a transgender sample, the association between gender non-conformity and experiences of being bullied was particularly strong among birth-assigned boys (Gower et al., 2018). In summary, it seems that transgender youth as a whole are bullied more often than their cisgender peers and that among transgender populations bullying is more common among those who present as gender non-conforming.

The association between gender non-conformity and being bullied may originate from heterosexism, a phenomenon describing the effort to govern traditional masculine and feminine roles in society based on the assumption that heterosexuality is the superior sexual orientation and the norm (Chesir-Teran, 2003; Toomey et al., 2012). In the same vein, the stronger association of perceived gender non-conformity and being bullied among natal males could be explained by males’ stronger tendency to safeguard traditional masculine roles (van Beusekom et al., 2020). Behavior deviating from culturally accepted masculine norms in boys is less readily tolerated than behavior deviating from the expected feminine behavior in girls (Ristori and Steensma, 2016). Even though the status of sexual and gender minorities has recently improved in many countries, heterosexism is widespread (Dunn and Szymanski, 2017), thus adolescents not conforming to gender norms may be more susceptible to bullying and harassment than their heterosexual gender-conforming peers.

However, confounding by internal stress needs to be considered when evaluating associations between transgender identity and being bullied. Gender minority stress and resilience (GMSR) theory (Hendricks and Testa, 2012; Testa et al., 2015) posits that gender minority people experience external stress, such as discrimination and victimization (such as being bullied), but also internal stress related to internalized transphobia and perceived stigma that predispose them to being constantly vigilant and anticipating discrimination. This may predispose to the development of depressive or hostile attribution bias (Morris, 2007; American Psychological Association, n.d.), possibly leading to the perception of victimization by peers when none was actually intended. Internal stressors may also include concealment of one’s identity. Although hiding one’s identity may reduce direct targeting by bullies, it may in turn create stress through identity non-affirmation and expose to mental distress such as depression, known indeed to be associated with transgender identity (Kaltiala-Heino et al., 2018). Mental health problems may in turn further induce negative attribution bias and experiences of being bullied and ostracized (Kaltiala-Heino and Fröjd, 2011). Therefore, when studying associations between gender identity and being bullied, the role of mental distress needs to be accounted for in order to reveal possible independent associations between transgender identity and being bullied. The role that transgender identity *per se* has in being bullied is important for school policies to tackle bullying, and for health and social policies.

Further, being bullied is commonly associated with perpetrating bullying (Cook et al., 2010; Shetgiri et al., 2012; Chan and Wong, 2015b). Those victimized themselves may reactively bully others or perpetrating bullying may be a way of defending oneself. On the other hand, aggressors often socialize in antisocial groups where delinquency occurs, thus elevating the likelihood of being victimized themselves (Jennings et al., 2012). Perpetrating bullying may therefore arise from having been victimized or vice versa.

Thus, when studying the role of gender identity in being bullied among adolescents, perpetrating bullying needs to be controlled for. Additionally, possible participation as a bully is an important problem in itself. To the best of our knowledge, the research so far has not explored bullying perpetrated among gender minority youth (McKay et al., 2019). However, Dank et al. (2014) found that transgender youth reported some of the highest perpetration rates of sexual harassment perpetration. As bullying among adolescents often has a sexual and heterosexist nature (Ashbaughm and Cornell, 2008; Toomey et al., 2012), similar associations might be expected with bullying perpetration. Elevated rates of bullying perpetration have also been found among sexual minority populations in some studies (Berlan et al., 2010; Eisenberg et al., 2015), who appear similar to transgender youth when it comes to being bullied.

To summarize, it appears that transgender youth are victims of bullying more commonly than their cisgender peers, but research has not taken account of confounding by perpetrating bullying or mental health factors (Day et al., 2018; Gower et al., 2018; Eisenberg et al., 2019; Johns et al., 2019; McKay et al., 2019; Bishop et al., 2020; Lowry et al., 2020; van Beusekom et al., 2020). The possible associations between transgender identity and perpetrating bullying are not known, leaving the understanding of the associations between gender identity and this common problem incomplete. According to the research so far, it moreover remains unclear whether involvement in bullying is similar across various gender minority identities or if opposite sex and non-binary identities differ in this respect. Additionally, most of the literature on transgender youth and bullying originates from North America, a possibly culturally different setting from Northern Europe. In this context, we ask and aim to answer the following questions:

(1) Is transgender identity associated with being bullied even when known correlates of involvement in bullying are controlled for?
(2) Is transgender identity associated with perpetrating bullying even when known correlates of involvement in bullying are controlled for?
(3) Are the possible associations similar between opposite sex identifying and non-binary youth?

During adolescence, small differences in age may have a large impact on development (Laursen and Hartl, 2013; Dahl et al., 2018). Involvement in bullying decreases as adolescents grow older (Boulton and Underwood, 1992; Liang et al., 2007), and with maturation of sexuality (Cacciatore et al., 2019) and identity development (Kroger et al., 2010), both older transgender youth and their mainstream peers are likely more confident and more able to handle diversity, which will likely also reduce involvement in bullying among transgender youth. Thus, we finally ask:

(4) Are these associations similar among middle and late adolescents?

We first expect to see that transgender adolescents report being bullied in excess in comparison to their cisgender peers, but that the associations will grow weaker when confounding by mental health correlates of bullying involvement and being a bully perpetrator are controlled for. Second, we hypothesize that transgender youth will also report more perpetration of bullying than their cisgender peers. Third, in line with heteronormative social control, we expect to see that the associations between gender identity and being bullied will be the strongest among non-binary/gender non-conforming youth. And finally, we expect to find that the associations between transgender identity and involvement in bullying will be weaker among older adolescents.

## Materials and Methods

### The School Health Promotion Study

The School Health Promotion Study (SHPS) of the National Institute for Health and Welfare is a school-based cross-sectional anonymous survey designed to examine the health, health behaviors, and school experiences of teenagers. The survey questionnaire is sent to every municipality in Finland. The municipalities decide if the schools in their area will participate in the survey and the vast majority of schools do indeed participate. The survey is run primarily for health policy and administrative purposes, and the data is available on request for purposes of scientific research. The main aim of the survey is to produce national adolescent health indicators that municipalities can utilize in planning services and that can be used at national level to assess the effectiveness of health policies. The authors obtained permission to use the data for scientific research but were not responsible for collecting it. The School Health Promotion Study has received ethical approval from Tampere University Hospital ethics committee and the ethics committee of the National Health Institute.

The survey is conducted among 8th and 9th graders of comprehensive school and second-year students in upper secondary education (upper secondary school and vocational school) which follow completion of the 9 years of comprehensive school. Survey participants in 2017 numbered 139,829. Of these, 48.9% (68,333) reported that they were male and 50.4% (70,539) that they were female. Of all respondents, 0.7% (957) did not report their sex, and these were excluded from further analyses. Of the respondents, 52.7% were in comprehensive school grades 8 or 9, 25.0% were attending upper secondary school, and 23.3% vocational school. The age of respondents in the comprehensive school sample was [mean (SD)] 14.83 (0.82) years, those in upper secondary school 16.84 (0.83) years and those in vocational school 17.29 (2.43) years. Of the respondents, 3.5% (*n* = 4,940) reported that they were 21 years old or older. These were excluded from further analyses. Descriptive information of the sample is given in Table 1. See section “Implausible, Likely Facetious Responding” for final sample size.

**TABLE 1:** Descriptive statistics.

	Comprehensive education		Upper secondary education	
		
Demographic variables	N (%)	M (SD)	N (%)	M (SD)
**Sex**				
Girl	36 123 (51.3%)		30 453 (50.8%)	
Boy	34 276 (48.7%)		29 520 (49.2%)	
Age		14.83 (0.82)		17.94 (2.17)
**Mother’s education**				
Only basic	3 815 (6.0%)		2 938 (5.2%)	
Other	59 705 (94.0%)		53 580 (94.8%)	
**Father’s education**				
Only basic	5 520 (8.9%)		5 191 (9.4%)	
Other	56 813 (91.1%)		50 315 (90.6%)	
**Family structure**				
Nuclear family	47 039 (69.5%)		38 699 (65.9%)	
Other	20 682 (30.5%)		20 053 (34.1%)	
**At least one parent unemployed in past 12 months**				
Yes	20 736 (31.0%)		18 384 (31.5%)	
No	46 229 (69.0%)		39 972 (68.5%)	
**Difficulties to communicate with parents**				
Yes	4 902 (7.3%)		3 713 (6.4%)	
No	61 946 (92.7%)		54 671 (93.6%)	
**Drinking alcohol weekly**				
Yes	2 790 (4.1%)		5 847 (9.9%)	
No	65 843 (95.9%)		53 349 (90.1%)	
Depression*		3.0 (1.5)		3.0 (1.5)
GAD-7**		3.8 (4.7)		3.9 (4.6)
**Gender identity**				
Cisgender	66 687 (95.7%)		57 540 (96.5%)	
Opposite sex	504 (0.7%)		313 (0.5%)	
Non-binary gender	2 483 (3.6%)		1 792 (3.0%)	
**Bullied someone**				
Yes	1 717 (2.5%)		750 (1.3%)	
No	68 125 (97.5%)		58 884 (98.7%)	
**Been bullied**				
Yes	3 438 (4.9%)		1 093 (1.8%)	
No	66 631 (95.1%)		58 789 (98.2%)	

*Range of depression was 2–8. **Range of GAD-7 was 0–21. The GAD-7 items describe the most prominent diagnostic features of the DSM IV generalized anxiety disorder.

### Measures

#### Sex and Gender Identity

The respondents were first asked “What is your sex?” with response alternatives “boy” and “girl” in the comprehensive school survey, and “male”/“female” in the upper secondary education response forms. This was intended to elicit the respondent’s sex as noted in their identity documents and was the opening question of the whole survey. Later, in the section of the survey addressing health, respondents were asked about their perceived gender as follows: “Do you perceive yourself to be…,” with response options “a boy/a girl/both/none/my perception varies.” According to sex and perceived gender, the respondents were categorized into one of three gender identities: cisgender identity (indicated male sex and perceives himself as a boy, or female sex and perceives herself as a girl), opposite sex identification (male sex, perceives herself as a girl; or female sex, perceives himself as a boy), and other/non-binary gender identity (independent of sex: perceived to be both a boy and a girl, perceived to be neither a boy nor a girl, variable).

#### Bullying

Bullying or being bullied was elicited using two questions derived from a World Health Organization study on youth health (King et al., 1996). The questions are based on Olweus’ definition of bullying (Olweus, 1993) that have been widely accepted as a basis for bullying research. Bullying was first defined as follows: “We say a student is being bullied when another student (or group of students), say or do nasty things to him or her. It is also bullying when a student is being teased repeatedly in a way she or he does not like. But it is not bullying when two students of about the same strength quarrel or fight.” Respondents were then asked how frequently they had been bullied during the ongoing school term, and how frequently they had bullied others: many times a week, about once a week, less frequently, and not at all. In the analyses, responses to these questions were dichotomized to about once a week or many times a week (= frequently) vs. less frequently or not at all.

#### Internalizing and Externalizing Symptoms

Internalizing symptoms studied were depression and generalized anxiety. Depression was measured with two screening questions: “During the past month, have you often been bothered by feeling down, depressed, or hopeless?” (yes/no) and “During the past month, have you often been bothered by little interest or pleasure in doing things?” (yes/no). These two questions have shown good psychometric properties in detecting depression in adolescents (Richardson et al., 2010). In the analyses, a sum score of these items was used as continuous variable. Generalized anxiety symptoms were elicited by the GAD-7, a self-report questionnaire designed to identify probable cases of generalized anxiety disorder and to assess symptom severity. The GAD-7 items describe the most prominent diagnostic features of the DSM IV generalized anxiety disorder. The GAD-7 elicits how often, during the last 2 weeks, the respondent has been bothered by each of the seven core symptoms of generalized anxiety disorder. Response options are “not at all,” “for several days,” “for more than half the days,” and “nearly every day,” scored, respectively as 1, 2, 3, and 4. The GAD-7 has been shown to be a reliable and valid measure for detecting generalized anxiety disorder in primary care and general population (Tiirikainen et al., 2019). In the analyses the sum score of these seven items was used as continuous variable.

Externalizing behaviors were represented, in addition to perpetrating bullying, by frequent consumption of alcohol. Alcohol consumption was elicited as follows: “How often do you use even small amounts of alcohol, for example half a can of beer or more?” with response options “once a week or more often/once or twice a month/about once a month/less frequently/not at all.” In the analyses the responses were dichotomized to once a week or more often (= frequently) vs. all other alternatives.

#### Family Variables

Family variables used were mother’s and father’s education (basic education, i.e., comprehensive school) only vs. at least upper secondary education, family structure [living with both parents (= nuclear family) vs. any other family constellation], parental unemployment (none vs. one vs. both parents unemployed or laid off during past 12 months) and difficulties in parent-adolescent communication (never able to discuss important things with parents vs. can talk with parents at least sometimes). Family variables were controlled for because they have a strong association with involvement in bullying (Knaappila et al., 2018).

#### Implausible, Likely Facetious Responding

It has been demonstrated that some adolescents deliberately mispresent themselves in survey studies, exaggerating their belonging to minorities as well as their problem behaviors, symptoms, and psychosocial problems (Cornell et al., 2012; Robinson-Cimpian, 2014). Due to this, the proportion of those reporting belonging to minorities appears implausibly high, and associations between minority status and psychosocial problems are overestimated. In relation to gender identity, such overestimation may risk a perception in society that gender variant youth are victims rather than active subjects participating in building the contemporary adolescent community. Particularly in light of the excessive media coverage of gender identity issues (Marchiano, 2017), gender identity is likely to be a topic which tempts adolescents to give facetious responses.

Excluding respondents reporting unlikely combinations of extreme responses outside the focus of present interest on topics theoretically not related to the variables of interest for the actual study questions has been shown to be an appropriate method for controlling for such facetious responding (Robinson-Cimpian, 2014; Kaltiala-Heino and Lindberg, 2019). In line with this, respondents reporting implausibly young age for being enrolled in the grades studied (<13 years), implausible shortness or height (extreme outliers) or who were calculated to have extreme BMI (< 10 or > 40) or reporting both extremely poor hearing, sight and mobility were classified as mischievous responders (for a detailed description, see Kaltiala-Heino and Lindberg, 2019). Being classified as a mischievous respondent was strongly associated with reporting transgender identity in this data (Kaltiala-Heino and Lindberg, 2019). These respondents (2.7%) were excluded from further analyses. Thus, the data in the analysis was from 130,372 respondents, of whom 96.1% were classified with cisgender identity, 0.6% with opposite sex identification, and 3.3% with other/non-binary gender identity. Descriptive statistics of the variables are presented in Table 1.

### Statistical Analyses

Associations between gender identity and involvement in bullying were first studied using cross-tabulations with chi-square statistics. Logistic regression was used to study multivariate associations. Gender identity was used as the independent variable, with cisgender as the reference category. (1) being bullied and (2) bullying others were entered each in turn as the dependent variable. As covariates, in the first model age and sex were added, in the second model family characteristics were added and finally, in the third model, internalizing symptoms, externalizing behaviors, and involvement in bullying in the other role (as a bully when being bullied was studied, and vice versa) were added. Odds ratios (OR) with 95% confidence intervals (95% CI) are given. Due to the large size of the data we set the limit for statistical significance at *p* < 0.001. The analyses were run separately for the comprehensive school and upper secondary education groups.

## Results

### Prevalence of Involvement in Bullying

Overall, reported prevalence of experiences of being bullied was higher in the comprehensive school sample than in the upper secondary education sample (4.9% vs. 1.8%). Similarly, reported prevalence of bullying others was higher in the comprehensive school sample (2.5% vs. 1.3%) (Table 1).

Experiences of being bullied were most commonly reported by non-binary students, followed by opposite sex identifying and cisgender students in both samples (Table 2).

**TABLE 2:** Experiences of bullying and bullying others according to gender identity,% (n).

	Cisgender	Opposite sex	Non-binary gender	*p*
***Comprehensive education***				
Been bullied	4.3 (2 886)	12.8 (64)	16.5 (404)	<0.001
Bullied others	2.0 (1 356)	8.9 (44)	10.5 (256)	<0.001
***Upper secondary education***				
Been bullied	1.6 (899)	5.8 (18)	8.5 (152)	<0.001
Bullied others	1.0 (582)	4.9 (15)	7.9 (140)	<0.001

Prevalence of perpetrating bullying followed a similar pattern. Bullying others was most commonly reported by non-binary students, followed in both samples by opposite sex identifying and cisgender students (Table 2).

### Relationship Between Gender Identity and Being Bullied

Table 3 presents the associations between gender identity and being bullied in the comprehensive school and upper secondary education samples before and after controlling for relevant confounding. Among the comprehensive school sample, opposite sex identification first yielded over twofold odds while non-binary identity yielded over fourfold odds for being bullied (Table 3; Model 1^a*^). In the upper secondary education sample, a similar pattern emerged but with even stronger associations (Table 3; Model 1^b**^).

**TABLE 3:** Regression analysis of being bullied.

	Comprehensive education			Upper secondary education		
		
	Model 1^a^*	Model 2^a^	Model 3^a^	Model 1^b^**	Model 2^b^	Model 3^b^
						
	OR (95% CI)	OR (95% CI)	OR (95% CI)	OR (95% CI)	OR (95% CI)	OR (95% CI)
**Gender ID (ref. cisgender)**						
Opposite sex	**2.86 (2.02–4.04)**	**2.67 (1.89–3.82)**	1.66 (1.13–2.49)	**3.77 (2.10–6.78)**	**3.29 (1.82–5.94)**	2.13 (1.01–4.17)
Non-binary	**4.22 (3.67–4.86)**	**3.52 (3.04–4.06)**	**1.98 (1.69–2.32)**	**5.21 (4.14–6.55)**	**4.35 (3.44–5.49)**	**1.99 (1.50–2.62)**
Official gender female (ref. male)	**0.76 (0.72–0.85)**	**0.73 (0.68–0.80)**	**0.54 (0.50–0.60)**	**0.64 (0.55–0.74)**	**0.60 (0.52–0.69)**	**0.64 (0.54–0.75)**
Age	0.96 (0.90–1–03)	0.94 (0.88–1.01)	0.83 (0.78–0.91)	1.04 (0.95–1.13)	0.99 (0.90–1.08)	0.94 (0.85–1.03)
Mother only basic education (ref. other)		**1.22 (1.03.1.45)**	1.17 (0.97–1.40)		**1.82 (1.42–2.36)**	**1.58 (1.18–2.10)**
Father only basic education (ref. other)		1.10 (0.95-1.28)	1.01 (0.87-1.19)		**1.26 (1.00-1.58)**	1.18 (0.92-1.50)
Nuclear family (ref. no)		1.09 (0.99–1.19)	0.97 (0.88–1.06)		**1.19 (1.03–1.39)**	1.11 (0.95–1.30)
Parental unemployment (ref. no)		**1.36 (1.25–1.49)**	**1.22 (1.13–1.33)**		**1.35 (1.17–1.57)**	1.25 (1.07–1.47)
Communication difficulties with parents (ref. no)		**2.51 (2.24–2.82)**	**1.31 (1.15–1.49)**		**2.45 (2.01–3.02)**	1.37 (1.09–1.74)
Alcohol weekly (ref. no)			1.30 (1.10–1.55)			1.30 (1.05–1.60)
Depression (continuous)			**1.18 (1.14–1.23)**			**1.16 (1.08–1.24)**
GAD-7 (continuous)***			**1.08 (1.06–1.09)**			**1.07 (1.04–1.09)**
Bullied others at least once a week (ref. no)			**9.20 (7.96–10.64)**			**46.97 (37.84–58.31)**

Statistically significant values (p < 0.001) presented in bold face. *Estimates in unadjusted model: Opposite sex OR (95% CI) = 2.73 (CI 1.93–3.86), non-binary OR (95% CI) = 4.09 (3.55–4.70). **Estimates in unadjusted model: Opposite sex OR (95% CI) = 3.51 (1.96–6.30), non-binary OR (95% CI) = 5.01 (4.02–6.34). ***Range of GAD-7 was 0–21. The GAD-7 items describe the most prominent diagnostic features of the DSM IV generalized anxiety disorder.

In each model presented in Table 3, more covariates were added and controlled for. The associations between opposite sex identification and non-binary identity with being bullied grew stronger when age and sex were added into the model but diminished when confounding by family variables, alcohol consumption, and finally mental health variables and perpetrating bullying were controlled for. Nevertheless, in the final model (Table 3; Model 3^a^), after controlling for the aforementioned covariates, a statistically significant association between non-binary identity and being bullied persisted among the comprehensive school sample [OR (95% CI) = 1.98 (1.69–2.32), *p* = 0.000]. Among the upper secondary education sample, the association likewise only persisted among non-binary youth [OR (95% CI) = 1.99 (1.50–2.62), *p* = 0.000] (Table 3; Model 3^b^).

Notably, the final models also revealed a strong correlation between being bullied and bullying others, particularly in the upper secondary education sample (comprehensive education sample, [OR (95% CI) = 9.20 (7.96–10.64), *p* = 0.000]; upper secondary education sample, [OR (95% CI) = 46.97 (37.84-58.31), *p* = 0.000] (Table 3; Models 3^a^ and 3^b^).

### Other Correlates of Being Subjected to Bullying

In addition to perpetrating bullying, certain family variables, depression, and anxiety were positively associated with being bullied among both samples. In both samples, a negative association emerged between natal female sex and being bullied (Table 3; Models 3^a^ and 3^b^).

### The Relationship Between Gender Identity and Perpetrating Bullying

Table 4 presents the association between gender identity and perpetrating bullying among the comprehensive school and upper secondary education samples before and after controlling for confounding. Throughout our models, the associations between gender minority identities and perpetrating bullying were stronger than the associations between gender minority identities and being bullied.

**TABLE 4:** Regression analysis for perpetration of bullying.

	Comprehensive education			Upper secondary education		
		
	Model 1^a^*	Model 2^a^	Model 3^a^	Model 1^b^**	Model 2^b^	Model 3^b^
						
	OR (95% CI)	OR (95% CI)	OR (95% CI)	OR (95% CI)	OR (95% CI)	OR (95% CI)
**Gender ID (ref. cisgender)**						
Opposite sex	**6.13 (4.09–9.19)**	**5.56 (3.69–8.39)**	**3.91 (2.47–6.19)**	**5.62 (2.85–11.12)**	**5.03 (2.54–9.99)**	3.15 (1.38–7.19)
Non-binary	**6.00 (4.98–7.24)**	**4.83 (3.98–5.86)**	**2.58 (2.07–3.21)**	**8.61 (6.71–11.05)**	**7.24 (5.60–9.35)**	**4.01 (2.91–5.52)**
Official gender female (ref. male)	**0.28 (0.25–0.32)**	**0.26 (0.23–0.30)**	**0.25 (0.21–0.29)**	**0.18 (0.14–0.23)**	**0.17 (0.13–0.21)**	**0.19 (0.15–0.25)**
Age	**1.13 (1.03–1.24)**	**1.09 (1.00–1.20)**	1.04 (0.94–1.15)	1.07 (0.96–1.20)	1.02 (0.90–1.14)	0.96 (0.84–1.08)
Mother only basic education (ref. other)		1.36 (1.07–1.72)	1.24 (0.96–1.59)		**2.01 (1.45–2.78)**	**1.51 (1.01–2.21)**
Father only basic education (ref. other)		1.20 (1.06–1.36)	1.29 (1.03–1.60)		1.12 (0.84–1.50)	0.96 (0.69–1.34)
Nuclear family (ref. no)		1.21 (1.06–1.38)	1.12 (0.98–1.28)		1.13 (0.94–1.37	1.05 (0.85–1.29)
Parental unemployment (ref. no)		1.21 (1.06–1.37)	1.08 (0.94–1.23)		1.17 (0.98–1.41)	1.03 (0.84–1.27)
Communication difficulties with parents (ref. no)		**2.71 (2.28–3.21)**	**1.59 (1.31–1.92)**		**2.62 (2.03–3.38)**	**1.83 (1.35–2.49)**
Alcohol weekly (ref. no)			**3.99 (3.34–4.77)**			**2.85 (2.28–3.56)**
Depression (continuous)			1.02 (0.95–1.08)			0.96 (0.87–1.06)
GAD-7 (continuous)***			**1.04 (1.02–1.06)**			1.03 (0.99–1.06)
Been bullied at least once a week (ref. no)			**8.90 (7.68–10.30)**			**45.68 (36.73–56.82)**

Bold face indicates statistically significant values (p < 0.001). *Estimates in unadjusted model: Opposite sex OR (95% CI) = 4.77 (3.21–7.10), non-binary OR (95% CI) = 5.10 (4.2–6.13). **Estimates in unadjusted model: Opposite sex OR (95% CI) = 4.31 (2.20–8.44), non-binary OR (95% CI) = 7.57 (5.93–9.66). ***Range of GAD-7 was 0–21. The GAD-7 items describe the most prominent diagnostic features of the DSM IV generalized anxiety disorder.

Initially in the comprehensive school sample, all transgender youth had over fourfold odds for perpetrating bullying (Table 4; Model 1^a*^). In comparison to the comprehensive school sample, the odds for perpetrating bullying were lower for the opposite sex identifying and higher for the non-binary identifying youth in the upper secondary education sample (Table 4; Model 1^b*^).

The associations between opposite sex identification and non-binary identity with perpetrating bullying grew stronger when age and sex were added into the model but diminished when confounding by family variables, alcohol consumption, and finally mental health variables and perpetrating bullying were controlled for.

In the comprehensive school sample, the association between gender identity and perpetrating bullying nevertheless persisted as statistically significant among both opposite sex identifying [OR (95% CI) = 3.91 (2.47–6.19), *p* = 0.000] and non-binary youth [OR (95% CI) = 2.58 (2.07–3.21), *p* = 0.000] although the association was stronger among opposite sex identifying youth (Table 4; Model 3^a^). In the upper secondary education sample, the association persisted statistically significant only among non-binary youth [OR (95% CI) = 4.01 (2.91–5.52), *p* = 0.000] (Table 4; Model 3^b^).

Notably, the final models also revealed a strong association in both samples between being bullied and bullying others [comprehensive education sample, OR (95% CI) = 8.90 (7.68–10.30), *p* = 0.000; upper secondary education sample, OR (95% CI) = 45.68 (36.73–56.82), *p* = 0.000]. The association was stronger among the upper secondary education sample (Table 4; Models 3^a^ and 3^b^).

### Other Correlates of Perpetrating Bullying

Positive associations between perpetrating bullying and difficulties communicating with parents and weekly alcohol consumption were found in both samples. In the comprehensive school sample, anxiety (but not depression) was also positively associated with perpetrating bullying. In both samples, a negative association between natal female sex and perpetrating bullying was found (Table 4; Models 3^a^ and 3^b^).

## Discussion

In this study we analyzed the association of gender minority identity with involvement in bullying among a large population-based sample of adolescents. We analyzed whether the association of gender identity and involvement in bullying differed among opposite sex and non-binary identifying youth or among middle and late adolescents.

We firstly found that in our large, nationally representative sample, being bullied was generally associated with transgender identity, and with non-binary identity in particular. This finding is in line with the existing literature, which indicates that experiences of being bullied are more common among gender minority than mainstream youth (Day et al., 2018; Eisenberg et al., 2019; Johns et al., 2019; Bishop et al., 2020). Various factors could explain this disparity. Transgender youth may differ from their peers in that their behavior or appearance deviates from traditional feminine and masculine roles. This could partly explain elevated rates of being bullied as bullying is often targeted at those perceived to deviate from the mainstream (Jones et al., 2018; Price-Feeney et al., 2018). More specifically, relating to sexual orientation and gender identity, bullying sexual and gender minorities could also stem from heterosexism, which refers to efforts to maintain traditional masculine and feminine roles in society (Chesir-Teran, 2003; Toomey et al., 2012). On the other hand, internal stress, as described in gender minority stress and resilience theory (Hendricks and Testa, 2012; Testa et al., 2015), could result in constant vigilance and anticipation of being victimized through the development of hostile or depressive attribution bias thus predisposing transgender youth to detect victimization by their peers where none was actually intended.

Secondly, we found that transgender identity was generally associated with perpetrating bullying and that the association was stronger than that of transgender identity and being bullied. To the best of our knowledge, past research has not examined perpetration of bullying among gender minority youth, thus rendering comparisons to prior research impossible. In a study by Dank et al. (2014), however, it was reported that the few transgender young people in their study were the ones most likely to perpetrate dating violence among their sample.

Such aggressive behavior could arise from being victimized or having witnessed victimization of other gender or sexual minorities (Eisenberg et al., 2016), as a coping mechanism or avenue through which one could release negative feelings.

On the other hand, adolescence in general is a mentally challenging time (Paus et al., 2008) during which adolescents struggle with a series of developmental tasks such as forming peer relations and coming to grips with their sexuality (Havighurst, 1948; Seiffge-Krenke and Gelhaar, 2008). The added complexity due to the emergence and further development of transgender identity could cause extra stress for adolescents. In this context, perpetrating bullying could be seen as sign of acting out, perhaps due to transgender adolescents’ own unresolved developmental issues.

Thirdly, non-binary identity was more strongly associated with involvement in bullying than opposite sex identity. Past research has found elevated rates of being subjected to bullying among youth (Lowry et al., 2020; van Beusekom et al., 2020) and transgender youth (Gower et al., 2018) who perceive themselves as more gender non-conforming (i.e., masculine females or feminine males) than youth with no such perception. Non-binary identifying youth particularly may display gender expression that does not conform to either masculine or feminine roles, and this may make them vulnerable to being bullied either due to simply being different from the mainstream, or as a result of heterosexist control. We found, however, that not only being bullied but also engaging in bullying was even more common among non-binary (perception of gender conforms to both or neither sex or it varies) than among opposite sex identifying youth.

It may be that the process of gender identity formation is a more complex process among non-binary youth than those young people identifying with the opposite sex. Such differences could stem from the nature of non-binary identity itself, as perceived gender may fluctuate, or align with both or neither traditional gender roles. This could delay the achievement of so-called transgender identity milestones, or factors associated with the formation of transgender identity, such as first living in the gender role felt within (Wilkinson et al., 2018) as young people struggle with their still unresolved gender identity. This internal turmoil due to uncertainty about one’s own identity, could, for example, impede the formation of peer relationships, a key part of adolescent development (Laursen and Hartl, 2013). This could exacerbate internal stress and predispose non-binary youth to mental health symptoms such as depression, which are known to relate to involvement in bullying (Kaltiala-Heino and Fröjd, 2011).

Finally, regarding age differences, the existing literature shows that as adolescents mature and progress toward adulthood, involvement in bullying decreases (Boulton and Underwood, 1992; Liang et al., 2007; Coulter et al., 2018). In line with this, involvement in bullying in our data was reported less commonly by the older adolescents in the upper secondary education sample across all gender identities. The association between opposite sex identification and being bullied also leveled out when confounding was controlled for in both samples. However, regardless of lower reported prevalence, the association between non-binary identity and perpetration of bullying was stronger among the older than among the younger adolescents in our study. It might be that those adolescents who still remain involved in bullying at an older age represent adolescents with the most developmental challenges. This finding could be seen to lend support to the notion that among transgender youth the possibly more complex nature of non-binary identity (in comparison to opposite sex identifying or cisgender youth) is indeed related to additional developmental challenges.

Additionally, while involvement in bullying was less prevalent among the older students of our study, the correlation between being bullied and being a bully grew stronger. This is likewise in agreement with the assumption that when involvement in bullying becomes less common as age increases, those who remain involved likely represent adolescents with the most developmental challenges. Being both a bully and a victim (bully-victim) is known to correlate with greatest amount of mental health problems and developmental difficulties (Forero et al., 1999).

### Strengths and Weaknesses of the Present Study

Our study has several strengths. Our large sample was an unselected, population-based sample representative of Finnish middle and late adolescents. This enhances the generalizability of our results.

There are indications even between European countries of variation in transgender youth’s peer relationships and psychological functioning (de Graaf et al., 2018; van der Star et al., 2018). One could speculate that such differences are even greater between European and North American adolescents. As most research on gender identity and involvement in bullying originates in the United States, we feel our study in a Northern European setting is a useful addition to the existing literature on the important subject of involvement in bullying and transgender identity.

We controlled in our analyses for a wide range of confounding factors closely related to involvement in bullying and gender minority identity. This allowed us to examine more closely the relationship between transgender identity and involvement in bullying. This is a strength of our study.

As has been recommended (Reisner et al., 2014; Eisenberg et al., 2017), we identified transgender youth with two separate questions located far apart from each other in the study questionnaire (“two-step method”). Due to the large sample size, we were additionally able to separate opposite sex identifying youth from non-binary youth, rather than grouping all transgender youth as one in our analyses.

Involvement in bullying was elicited using questions derived from WHO’s Youth Study (King et al., 1996). The WHO questions have since then been used in numerous studies across countries (for review see Kaltiala-Heino and Fröjd, 2011) which makes data elicited with them comparable with earlier research. This is a strength of our study.

Our study also has several weaknesses. In spite of our large sample, the number of transgender youth reporting perpetrating bullying was on the smaller side, although we still feel we reached adequate cell sizes for statistical validity.

In the present study, a secondary data was used. The data was not planned nor collected by us, and we were therefore unable to influence the way certain topics of interest were elicited. As a result, the way experiences of bullying were elicited in the study questionnaire made it impossible to distinguish between different types of bullying behavior in which adolescents had been involved, such as traditional school bullying or cyberbullying, or physical and verbal bullying and exclusion.

Additionally, whether respondents were living in their desired gender roles was not elicited in the questionnaire. This inhibited additional comparisons regarding involvement in bullying among those who conceal their gender identity vs. those who do not. The GMSR theory suggests concealment of one’s experienced gender identity (for example not living in the desired gender role) is a stressor that could possibly negatively affect mental health of gender minority people. One could thus speculate that living in the desired gender role could in fact reduce mental health symptoms such as depression, thus decreasing bullying involvement, a behavior associated with mental health issues. On the other hand, living in the desired gender role could manifest as behavior or appearance deviating from traditional masculine and feminine roles (such as natal girls using boys’ restrooms or natal boys having a more feminine appearance) thus predisposing youths to bullying, a behavior commonly directed to those who deviate from the mainstream. Lastly, as the study was a cross-sectional one, caution must be exercised when interpreting the results as causality cannot be determined from such data.

## Conclusion

Transgender identity and non-binary identity in particular, is associated with both being bullied and bullying others even when a range of variables, including internal stress and involvement in bullying in the opposite role, are taken into account. This could suggest that the development of transgender identity (and non-binary identity in particular) is an additional stress for youth as they navigate the already developmentally challenging years of adolescence toward adulthood.

Future studies should focus on including gender minority specific measures in study questionnaires. Such measures could include various gender identities and for example gender minority specific stressors named in the GMSR theory, such as living in the desired role. Such measures could help uncover in more detail the association between bullying involvement and various gender identities *per se*.

Programs that promote gender diversity should be implemented in schools and in larger context in the society with the aim of reducing heteronormativity and promoting the acceptance of gender diversity.

Teachers, parents and health care workers must consider that gender minority youth are not necessarily only victims but also perpetrators of bullying.

## Data Availability Statement

The data analyzed in this study is subject to the following licenses/restrictions: The data belongs to the Finnish Institute for Health and Welfare and is available for researchers by application. Requests to access these datasets should be directed to pauliina.luopa@thl.fi.

## Ethics Statement

The studies involving human participants were reviewed and approved by the Tampere University Hospital ethics committee (Tampere University Hospital, Tampere, Finland) and National Institute of Health and Welfare ethics committee (National Institute of Health and Welfare, Helsinki, Finland). Written informed consent from the participants’ legal guardian/next of kin was not required to participate in this study in accordance with the national legislation and the institutional requirements.

## Author Contributions

RK conceived the idea of the study and supervised the project. NE and RK designed the statistical analyses while NE carried them out. EH wrote the manuscript with help from RK and NE. All authors contributed to the final manuscript, each with a specific focus.

## Conflict of Interest

The authors declare that the research was conducted in the absence of any commercial or financial relationships that could be construed as a potential conflict of interest.
